# Rehabilitation of hemineglect of the left arm using movement detection bracelets activating a visual and acoustic alarm

**DOI:** 10.1186/s12984-016-0191-0

**Published:** 2016-09-02

**Authors:** Jose M. Trejo-Gabriel-Galan, V. Rogel-Melgosa, S. Gonzalez, J. Sedano, J. R. Villar, N. Arenaza-Basterrechea

**Affiliations:** 1 Hospital Universitario de Burgos, Ave. Islas Baleares 3, 09006 Burgos, Spain; 2 Instituto Tecnológico de Castilla y León, Burgos, Spain; 3 Computer Science Department, Universidad de Oviedo, Oviedo, Spain

**Keywords:** Hemisensory neglect, Neglect rehabilitation, Fuzzy logic, Activities of daily living, Medical device

## Abstract

**Background:**

Hemineglect is frequent after right hemisphere stroke and prevents functional independence, but effective rehabilitation interventions are lacking. Our objective was to determine if a visual-acoustic alarm in the hemineglect arm activated by a certain discrepancy in movement of both hands can enhance neglect arm use in five tasks of daily living.

**Methods:**

In this pre-post intervention study 9 stroke patients with residual hemineglect of the arm were trained for 7 days in five bimanual tasks of daily living: carrying a tray, button fastening, cutting food with knife and fork, washing the face with both hands and arm sway while walking. This was done through motion sensors mounted in bracelets on both wrists that compared movement between them. When the neglect-hand movement was less than a limit established by two fuzzy logic based classifiers, a visual-acoustic alarm in the neglect-hand bracelet was activated to encourage its use in the task.

**Results:**

Both motion and function of the neglect hand improved during the seven days of training when visual-acoustic alarms were active but a worsening to baseline values occurred on day 8 and day 30 when alarms where switched off. Improvement was limited to vision-dependent tasks.

**Conclusions:**

Neglect-hand improvement with this approach is limited to bimanual activities in which an object is manipulated under vision control, but no short or long term learning happens.

## Background

In visual-spatial hemineglect (also known as hemi-inattention) patients with a lesion of the right cerebral hemisphere are not aware of objects in the left visual field despite not having a visual deficit. When it encompasses left limbs, as well as lacking awareness of them, the patient does not use the left arm in spite of not having paralysis. Neglect predicts not regaining functional independence [1]. In more than 85 % of patients with right hemispheric stroke, hemineglect is found in at least one pencil and paper tests such as cancellation of lines and marking lines in their middle point, copy of superimposed shapes or of a figurative drawing. But in 36 % of cases, neglect in activities of daily living cannot be detected by these tests [2]. Among the 28 standardized tests for hemineglect [3], the Catherine Bergego scale is one of the most used and asks about performance of the patient in activities of daily living but does not measure the performance itself. Several rehabilitation strategies for hemineglect have been used [4, 5] including forced visual sweep scanning, trunk rotation, application of muscle vibration in the neck, mental images, visual prisms, sensory activation of the left arm [6], vestibular stimulation on the left side, and transcranial magnetic stimulation [7]. Currently, there is insufficient evidence to recommend a particular rehabilitation strategy for neglect as shown by a Cochrane review that found no efficacy of rehabilitation interventions in reducing disability [8, 9]. In this pre-post intervention pilot study, we studied if a visual-acoustic alarm in the hemineglect arm activated by its reduced movement relative to the contralateral arm could increase neglect arm use in five tasks of daily living. To monitor arm movement, we used triaxial accelerometers, previously employed to measure upper limb movement after stroke [10, 11].

## Methods

Design: This is a pre-post intervention study in a consecutive, convenience sample of hemineglect patients.

Subjects: The study included 9 subjects (4 males and 5 females, aged between 65 and 85 years). All subjects had left hemineglect including the hand except one female that had right hemineglect. They had been consecutively admitted to the Neurology Department of “Hospital Universitario” in Burgos (Spain) for a contralateral stroke (8 of right and 1 of left hemisphere) that had damaged the cerebral cortex six or more months before the beginning of the study. For the sake of simplification, the single patient with a right arm neglect has been and analyzed with all the rest of the patients with left arm neglect. After a complete neurological examination, hemineglect of the arm was defined when there was extinction of tactile and visual simultaneous stimuli and data of neglect in the following tests: cancellation of lines, marking of lines at their middle point and cancellation of stars among distracting figures. Hemineglect was quantified by the Catherine-Bergego scale. Other inclusion criteria were a normal or near normal strength of the hand (4+ on the Medical Research Council scale), and preserved sight and hearing. Exclusion criteria were cognitive or language impairment, anesthesia of the arm in any sensory modality or any physical, psychological o social limitation for participating in the study or for follow-up in the opinion of the investigator. These exclusion criteria or refusal to participate prevented 11 other screened patients from entering the study. This study was approved by the “Hospital Universitario de Burgos” Clinical Trials and Ethics Committee and patients agreed their participation by signing an informed consent prior to their inclusion.

Devices: Motion sensors embedded in light bracelets for both wrists, included in the left a visual (flashing light) and audible (beep) alarm that was triggered and emitted its light and sound when the lack of left arm movement caused a certain asymmetry with the right arm movement. Sensors included a tri-axial capacitive accelerometer made of silicon by microelectronic system technology. It had a sampling frequency of 16 Hz, could store data in its memory during 45 min and when memory was filled, data could be transferred to a computer. The pair of sensors were synchronized using an “ad-hoc” 433 MHz wireless link between them, allowing an almost simultaneous gathering of samples. Data from each sensor was processed using a sliding window of 1 s length and no overlap, computing the Amount of Movement transformation. The mean and the maximum Amounts of Movement was finally determined using a second sliding window of size 10 and no overlap. Two Mamdani Fuzzy Rule Based Classifiers (FRBC) were used in order to determine the degree of dissimilarity among the movement of both hands: one FRBC took as inputs the mean Amount of Movement from both hands, while the other the maximum Amount of Movement. A fuzzy partition was proposed for each input fuzzy variable, with granularity 3 and Gaussian-type fuzzy membership functions. The output of both FRBC was also a fuzzy variable with granularity 3 as well, but with triangle-shaped membership functions. It could range from 0-100, higher values indicating more right-left hand motion dissimilarity. The parameter setting was performed with a genetic algorithm using a vector of real values individual representation, the blend-alpha cross-over operator with alpha set to 0.3 and a mutation operation with probability 0.02. Previous unpublished use of this model made us assign a threshold of dissimilarity of 40 for normal and of 60 for hemineglect subjects, above which the alarm would set off. The hemineglect patients repeated during one hour the five bimanual tasks mentioned below during days 1 to 7 following the instructions of one of the authors: an occupational therapist (R-M, V) with experience in rehabilitation of activities of daily living. Patients were encouraged to use the left arm as much as the right arm in the five bimanual tasks to avoid movement dissimilarity between both hands that would set off the alarm. The intervals analyzed to set off an alarm were of 10 s; the audio-visual alarm stopped when the patient corrected the movement or after 10 s without correction. If there was no correction, it stopped for 3 s and was ready to be triggered again.

Variables: five bimanual tasks of daily living were evaluated with the motion sensors previously described: two intended to be representative of tasks in which both hands are used as a whole (hand sway while walking, simulating washing the face with both hands) and other three of more variable tasks, with need for visual control and for adjustment of movements during the activity (carrying a tray, fastening buttons and cutting food with knife and fork).

The following two efficacy variables measured the performance of the left hemineglect hand using the contralateral right hand as control in each of these tasks. The first variable was a measure of movement dissimilarity between hands, was calculated subtracting the right from the left hand movement data registered by motion sensors, and was named “asymmetry of hand movement”. A decrease in asymmetry indicated a better performance.

The other was a surrogate variable for “functional improvement” and consisted of the number of times that the improvement allowed making the alarm trigger more demandingly while performing the five mentioned tasks from the mentioned initial 60 threshold level. Two points would be reduced the next exercise day when at the end of the actual session the alarm had set off less than half the number of times the previous day, and four points when the alarm had not set off at all.

Data collection: During the measurements, the previously mentioned bimanual five tasks were done in the same order and had the same duration every day. Although sensors registered movement data every day, on certain days visual-acoustic alarms were silenced: on day 0 to register baseline data and on days 8 and 30 to compare captured data with that of day 0. This was done to assess if the exercise of activities during days 1 to 7 based in the active visual and acoustic alarm feedback improved movement persistently in the absence of alarms.

Statistical analysis: All analysis was performed for each of the five above mentioned tasks in days 0, 1, 7, 8 and 30. Description of data was followed by statistical analysis of both efficacy variables “asymmetry of movement” and “functional improvement” variables. Due to the non-normal distribution of all variables and sample size, nonparametric tests were chosen: Wilcoxon rank sign test for repeated measures or the Friedman test depending on whether two or more assessments of the efficacy variable were done, and the Kruskal-Wallis test in case of no repeated assessments of the variables. A 95 % confidence interval or *P* < 0.05 defined statistical significance. The statistical package for social science (SPSS v. 19) was used.

## Results

In each of the five tasks, the number of alarms and trigger modifications allowed by improvements in performance were distributed differently (Kruskal-Wallis, *p* <0.002), indicating that motor performance in the tasks was heterogeneous, as expected. In all cases the hand suffering hemineglect moved less than the contralateral, healthy hand-according to the definition of hemineglect-, producing an asymmetry in hand motion. This asymmetry decreased during day 1 to 7 in which activities were exercised with alarm feedback, in some cases to the point of reverting (Table 1). The other efficacy variable (“functional improvement”) also ameliorated in all five tasks, which allowed resetting the alarm trigger at least in one of days 2-7 and in three days in the activity of cutting food with knife and fork.

**Table 1:** Evolution of asymmetry of movement between right and left (R-L) hand at baseline (not emitting audio and visual alarms), mean asymmetry during seven days of training (with audio and visual alarms activated), number of times that alarms were triggered and the mean of the number of times in which improvement allowed a more demanding trigger of alarms

Task	Walking	Tray	Washing face	Button fastening	Knife and fork
Baseline R-L asymmetry of movement^a^	0.97	0.98	0.25	0.52	0.81
Days 1-7: Mean R-L asymmetry of movement^a^	0.61	−0.10	0.24	−0.15	−0.35
Days 1-7: mean number of alarms triggered^a^	9.97	7.79	16.48	33.97	11.41
Days 1-7: number of trigger difficulty increases	2.55	1.97	1.00	1.33	3.22

^**a**^A positive value indicates greater movement of the right hand, a negative value greater movement of the left hand

As shown in Table 2, in the five tasks as a whole there was a decrease of right-left hand movement asymmetry from baseline (without visual-acoustic alarm feedback) to both days 1 and 7 (with active visual-acoustic alarms), that is, there was a global improvement of left hand movement on days 1 and 7, associated with visual-acoustic alarm feedback. Analyzing each activity separately, this improvement on both days 1 and 7 was restricted to button fastening, using knife and fork, and carrying a tray but the use of the left hand was not improved for those activities done without visual control (i.e. face washing and left hand sway during walking). In the tasks of carrying a tray, fastening a button and using knife and fork Table 3 also shows global differences among asymmetries of right-left hand movement between all three days (on baseline, on day 1 and on day 7), but no differences are found between the three measurements with visual-acoustic alarms inactive (baseline, day 8 and day 30).

**Table 2:** Variation of right to left hand asymmetry of movement from baseline to day 1 and from baseline to day 7

	Set of tasks	Walking	Tray	Washing face	Button fastening	Knife and fork
Basal-day 1	0.000	0.25 (n.s.)	0.008	0.86 (n.s.)	0.008	0.008
Basal-day 7	0.000	0.66 (n.s.)	0.028	0.46 (n.s.)	0.028	0.028

Wilcoxon *T* signed-rank test. *P*-values are shown

**Table 3:** Global differences of right-left hand movement asymmetries between baseline, day 1 and day 7 and between baseline, day 8 and day 30

	Set of tasks	Walking	Tray	Washing face	Button fastening	Knife and fork
Baseline, day 1 and day 7	0.000	1.00 (n.s.)	0.009	0.85 (n.s.)	0.002	0.011
Baseline, day 8 and day 30	n.s.	n.s.	n.s.	n.s.	n.s.	n.s.

Friedman test. *P*-values are shown

**Fig. 1:**
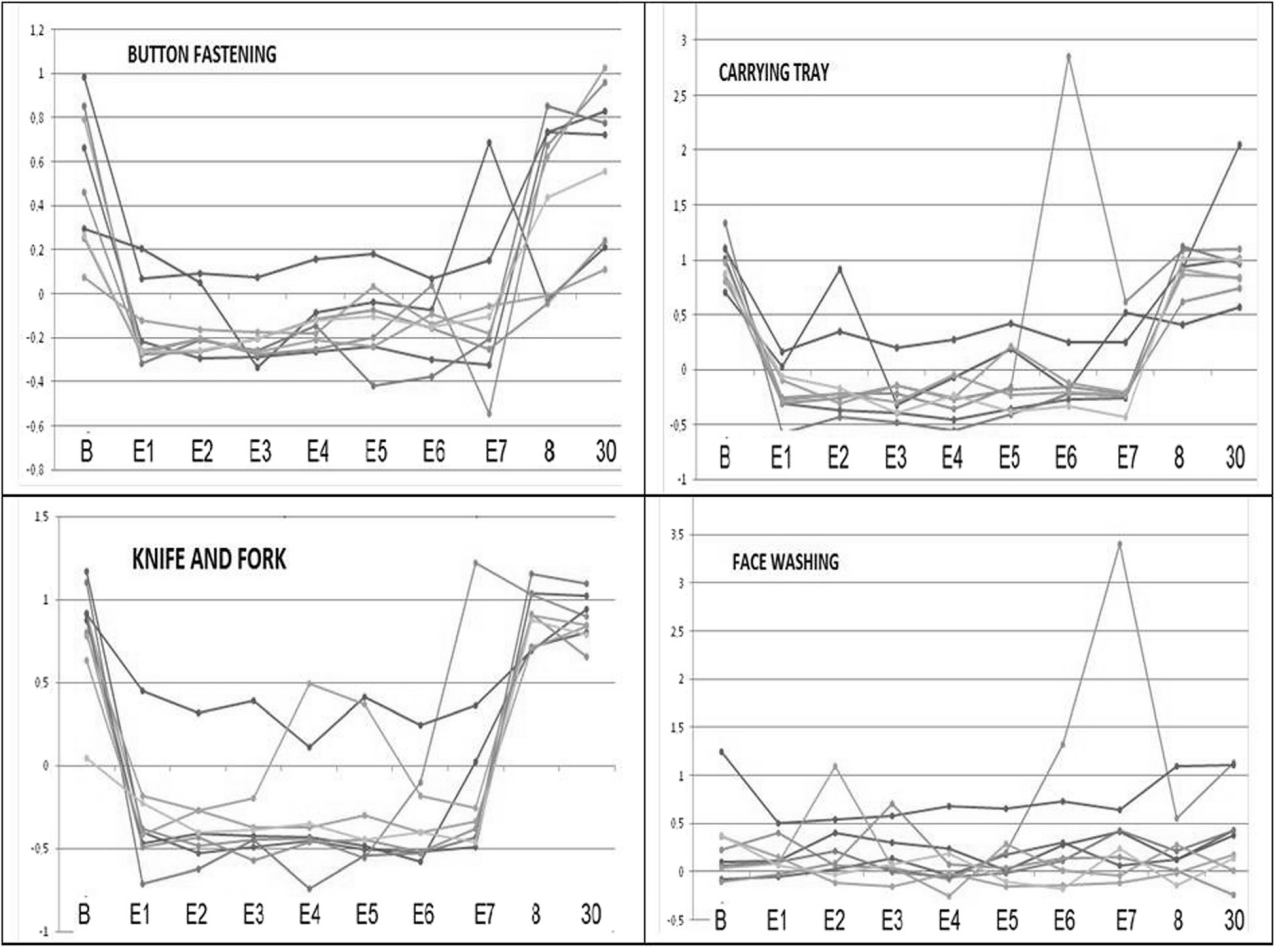
Asymmetry of motion of the 9 subjects between the right and left hands without visual-acoustic alarms [baseline (B), day 8 and day 30] and days 1 to 7 of exercise of activities (E1-E7) with visual-acoustic alarms active

Curves on Fig. 1 show the right-left hand motion asymmetry of the 9 subjects without visual-acoustic alarm feedback [baseline (B), day 8 and day 30] and with visual-acoustic feedback (days 1 to 7 of exercise of activities: E1-E7). In all activities except face washing and arm sway while walking (this last not shown) right to left hand movement asymmetry were reduced on day 1 when alarms were switched on and this held up to day 7 and there was a brisk asymmetry increase on days 8 and 30, when visual-acoustic alarms were again switched off.

## Discussion

The main finding of the study is an improvement of the left hand suffering neglect, both in movement and functionally, during tasks of daily living when analyzed globally and in particular in the following three: fastening buttons, carrying a tray and using fork and knife to eat. But this improvement only happened while the alarm emitted its visual-acoustic signals when triggered by a reduced movement of the left arm suffering neglect relative to the right arm. In this way, there was an improvement from baseline to day 1 and to day 7, of both efficacy variables while visual-acoustic alarms where active but not to day 8 and to day 30, when alarms were disconnected and the patient did not receive those sensorial feedbacks when underperforming with his/her left, neglect arm. It can be deduced that even when hemineglect is formally confirmed in every patient as was the case in this study, it is not complete as visual-acoustic stimuli can still increase motor behavior. Although visual hemineglect is much better known, right hemispheric lesions can also cause auditory neglect of acoustic stimuli within the left hemi-space [12]. If the improving feedback is visual, acoustic or both merits further study but we found the following hint of their relative importance in the two activities that could not receive the visual signal from the alarm. Unlike global findings, in face washing with the hands and arm sway while walking activities, left arm motion did not improve even while receiving the visual-acoustic feedback of the alarm. As these activities are carried out without visual input (eyes closed or not looking at the hands) it is suggested that auditory neglect is profound and prevents the alarm “beep” to enhance left arm motion. The apparently simple gesture of washing the face with both hands, but for which eyes are closed, is the second task triggering more alarms (after fastening buttons, a task with greater difficulty and that is sometimes performed without visual control) which suggest that also in visual hemineglect, when visual stimuli cannot be received motor performance suffers. Conversely, cutting food with knife and fork, the most visually dependent activity, is also the one in which more changes to a more demanding alarm trigger were possible, the surrogate measure of functional improvement that we established. Alternatively, face washing and hand sway activities could have a more “automatic” nature and therefore less dependence from external stimuli.

In inferior parietal lobule lesions, the perceptive component of hemineglect is more marked than in dorso-lateral prefrontal lesions, in which the motor component is more apparent, while deep lesions of the temporal lobe causes object focused hemineglect [13]. It is this last aspect of object focused hemineglect (cutting with knife and fork, fastening buttons, carrying tray), which improved more clearly in this study by the visual-auditory feedback and not purely motor hemineglect like arm swing when walking, or washing face tasks.

Figure 1 shows the neglect arm motion dependence on visual-acoustic alarms: improvement from baseline to day 1 when alarms were switched on (except in face washing and in arm sway while walking, this last not shown), but brisk worsening on day 8 and day 30, when they were switched off. The curves were flat from day 1 to day 7, indicating that the improvement was maintained but did not increase during the seven days of training, suggesting no learning during the training. Indeed, worsening on day 8 and day 30 shows that there was no posterior short term or lasting learning in the absence of visual-acoustic feedback provided by the alarm. This could be due either to insufficient duration of the training or to its inability to generate stimuli-independent neural circuits that can overcome neglect. This absolute dependency on feedback (visual-acoustic in this case) for the performance of motor activities is postulated by the *guidance hypothesis* of motor learning [14].

A pre-post intervention design without controls is a limitation that cannot exclude placebo effect, but we deem it unlikely, as some tasks were improved and others not at all by visual-acoustic alarms. Also, bracelets were worn in all evaluations, even when visual-acoustic alarms were disabled. A future study with more patients, with a control group, and using separate visual and acoustic alarms is needed after this preliminary study. There are also significant strengths in the presented data: it does not use neglect tests but tasks of daily living with efficacy variables being objective measures of motion and of function and processed by fuzzy logic, a branch of artificial intelligence appropriate to analyze real-world information, such as human motor activity recognition [15].

## Conclusions

A system of rehabilitation of arm neglect has been evaluated, consisting of a visual-acoustic alarm emitting signals when motion sensors mounted in wrist bracelets detected that the neglect arm lagged behind the right arm in five bimanual tasks of daily living. Data from motion sensors were analyzed using fuzzy logic based models. During the week in which visual-acoustic alarms were active, there was a stable improvement of left hand movement and function. This improvement was limited to tasks that need greater visual control and adjustments (cutting with knife and fork, buttoning and carrying a tray), that is, of hemineglect focused on an object. But there was no improvement in more “automatic” tasks without object manipulation or without the need of visual control (arm sway while walking, washing face). This improvement disappeared as soon as the visual-acoustic alarms were switched-off, suggesting no short nor long term learning.

## Abbreviations

FRBC: Fuzzy rule based classifier
